# Pore‐Size Distribution and Frequency‐Dependent Attenuation in Human Cortical Tibia Bone Discriminate Fragility Fractures in Postmenopausal Women With Low Bone Mineral Density

**DOI:** 10.1002/jbm4.10536

**Published:** 2021-09-02

**Authors:** Gabriele Armbrecht, Huong Nguyen Minh, Jonas Massmann, Kay Raum

**Affiliations:** ^1^ Charité—Universitätsmedizin Berlin, Corporate Member of Freie Universität Berlin, Humboldt‐Universität zu Berlin, and Berlin Institute of Health, Department of Radiology, Center for Muscle and Bone Research Berlin Germany; ^2^ Charité—Universitätsmedizin Berlin, Corporate Member of Freie Universität Berlin, Humboldt‐Universität zu Berlin, and Berlin Institute of Health, Center for Regenerative Therapies Berlin Germany

**Keywords:** CORTICAL BONE, FRACTURE DISCRIMINATION, CLINICAL TRIAL, DXA, QUANTITATIVE BONE ULTRASOUND

## Abstract

Osteoporosis is a disorder of bone remodeling leading to reduced bone mass, structural deterioration, and increased bone fragility. The established diagnosis is based on the measurement of areal bone mineral density by dual‐energy X‐ray absorptiometry (DXA), which poorly captures individual bone loss and structural decay. Enlarged cortical pores in the tibia have been proposed to indicate structural deterioration and reduced bone strength in the hip. Here, we report for the first time the in vivo assessment of the cortical pore‐size distribution together with frequency‐dependent attenuation at the anteromedial tibia midshaft by means of a novel ultrasonic cortical backscatter (CortBS) technology. We hypothesized that the CortBS parameters are associated with the occurrence of fragility fractures in postmenopausal women (*n* = 55). The discrimination performance was compared with those of DXA and high‐resolution peripheral computed tomography (HR‐pQCT). The results suggest a superior discrimination performance of CortBS (area under the receiver operating characteristic curve [AUC]: 0.69 ≤ AUC ≤ 0.75) compared with DXA (0.54 ≤ AUC ≤ 0.55) and a similar performance compared with HR‐pQCT (0.66 ≤ AUC ≤ 0.73). CortBS is the first quantitative bone imaging modality that can quantify microstructural tissue deteriorations in cortical bone, which occur during normal aging and the development of osteoporosis. © 2021 The Authors. *JBMR Plus* published by Wiley Periodicals LLC on behalf of American Society for Bone and Mineral Research.

## 1 Introduction

Osteoporosis (OP) is an age‐associated disorder of bone remodeling leading to reduced bone mass, structural deterioration, and increased bone fragility.^(^ ^1^ ^)^ Although OP is generally thought of as a “woman's disease,”^(^ ^2^ ^)^ men account for a third of OP‐related hip fractures in Europe.^(^ ^3^ ^)^ An estimated 1.0 million quality‐adjusted life years (QALYs) were lost in 2017 due to fragility fractures. According to a recent systematic review of burden and management of fragility fractures in the largest EU countries,^(^ ^4^ ^)^ fragility fractures are ranked number four among 16 common noncommunicable diseases with respect to the disability‐adjusted life years (DALYs). For individuals aged >50 years, DALYs were higher than those for stroke. Established guidelines for the diagnosis of OP recommend the assessment of fracture risk factors and the *T*‐score, which is derived from the measurement of areal bone mineral density (aBMD) by means of dual‐energy X‐ray absorptiometry (DXA) at major fracture sites, ie, spine and proximal femur.^(^ ^5^ ^)^ OP is defined for aBMD values 2.5 SD or more below the mean assessed in young adults (ie, *T*‐score ≤ −2.5). Low bone mass (osteopenia, −1 ≥ *T*‐score > −2.5) is currently not considered as a disease,^(^ ^5^ ^)^ although a specific osteoprotective therapy is recommended if additional clinical risk factors are present.^(^ ^6^ ^)^ However, bone strength is determined by a plethora of factors, including size, shape, architecture, and composition.^(^ ^7^ ^)^ Today, there is increasing evidence that the majority of individuals who have sustained an osteoporosis‐related fracture or who are at high risk of fracture are not diagnosed as osteoporotic according to the BMD level.^(^ ^8^, ^9^ ^)^

Bone tissue undergoes permanent remodeling. Under normal conditions, osteoclasts create resorption canals in the cortical bone tissue matrix, which are refilled by osteoblasts leaving a Haversian canal with a diameter of approximately 30 μm.^(^ ^10^ ^)^ Bone loss occurs in both women and men as part of the natural aging process.^(^ ^11^ ^)^ Unbalanced intracortical remodeling typically starts in the endosteal subcompartment and leaves partially refilled or even nonrefilled bone multicellular units (BMUs). Clustering of BMUs enhances their chances to merge, creating “giant” pores with diameters larger than 385 μm,^(^ ^12^ ^)^ leading to the so‐called trabecularized cortex^(^ ^13^ ^)^ and ultimately to a thinning of the cortical bone shell. In the femoral neck of elderly people (aged 57 to 98 years), decreases of cortical thickness (Ct.Th) and increases of porosity (Ct.Po) by ~4% and ~32%, respectively, per decade have been observed.^(^ ^14^ ^)^ In contrast, pore density (Ct.Po.Dn) was only reduced in the elderly (aged 87 to 98 years) compared with the middle‐aged group (aged 57 to 68 years).^(^ ^14^ ^)^ Postmenopausal women with osteopenia have higher cortical porosity and thinner cortices at the distal radius and tibia than women with normal aBMD.^(^ ^15^ ^)^ In an ex vivo study, cortical thinning and the prevalence of large BMUs in the tibia were found to be associated with structural deterioration of the femoral neck^(^ ^16^ ^)^ and proximal femur strength.^(^ ^17^ ^)^ Although cortical porosity of the tibia was not associated with femoral stiffness or strength, the proportion of Ct.Po attributable to large pores (diameter >100 μm) was significantly associated with hip strength in both standing (*r* = −0.61) and falling (*r* = 0.48) conditions. However, cortical thinning is partially compensated by the apposition of new tissue matrix at the periosteal interface leading to an increased bone diameter and a further increase of the pore diameter gradient in the radial bone direction. The theory showing that bending strength is largely dictated by the size of the largest pores has been proposed by Griffith^(^ ^18^ ^)^ and is nowadays well established in the field of fracture mechanics of cement‐based materials.^(^ ^19^ ^)^ Therefore, the cortical pore diameter distribution Ct.Po.Dm.D (hereinafter simply called pore‐size distribution) represents a relevant target for identifying patients with high fracture risk. However, more than 60% of intracortical pores are smaller than 100 μm in diameter.^(^ ^13^ ^)^ Given their small size, the in vivo imaging of cortical pores remains challenging, even with the most advanced high‐resolution peripheral quantitative computed tomography (HR‐pQCT). The imaging resolution of the first‐ and second‐generation HR‐pQCT systems with voxel sizes of 82 and 61 μm allows direct visualization and segmentation of large pores (ie, Ct.Po.Dm >100 μm) only, leaving the major fraction of smaller cortical pores unresolved. Iori and colleagues^(^ ^20^ ^)^ have proposed a calibration rule for the estimation of Ct.Po locally from volumetric BMD (vBMD) distribution parameters. This method is more accurate (absolute error 3.4%) than established vBMD or threshold‐based approaches, as it approximates the contribution of unresolved pores (ie, Ct.Po.Dm <80 μm). However, the HR‐pQCT technology is not widely distributed and is used mostly in clinical research so far. With DXA‐based aBMD, ie, the diagnostic gold standard, cortical bone loss resulting from structural decay is poorly captured.^(^ ^9^, ^21^, ^22^ ^)^

Quantitative ultrasound (QUS) methods are non‐ionizing alternatives for the diagnosis of osteoporosis and the prediction of fracture risk.^(^ ^23^ ^)^ Many QUS approaches target trabecular sites, eg, at the heel, and predicting BMD via empirical associations with the measured speed of sound (SOS), broadband ultrasound attenuation (BUA), and other parameters derived from the measurement.^(^ ^24^ ^)^ More recent QUS technologies, eg, bidirectional axial transmission, measure cortical sites, eg, distal radius and tibia, and aim at the quantitative assessment of structural cortical bone properties, eg, Ct.Th and Ct.Po.^(^ ^25^ ^)^ However, none of the existing diagnostic technologies can assess quantitative information about the cortical pore micromorphology. Particularly, the transition from a normal age‐ and sex‐specific pore‐size distribution to a pathologically altered one caused by large BMUs could not be assessed in vivo so far.

We have recently developed a theoretical cortical bone backscatter model (CortBS) and an ultrasonic multi‐angle 3D acquisition and data processing scheme to assess microstructural properties in cortical bone.^(^ ^26^ ^)^ The method measures the frequency‐dependent attenuation and backscatter coefficients *α(f)* and *BSC(f)* at the tibia and retrieves the cortical pore‐size distribution Ct.Po.Dm.D by fitting a theoretical backscatter coefficient to the measured *BSC(f)*. In an ex vivo study on bones from 19 human donors, pore‐size parameters, particularly those describing the prevalence of large pores, could be assessed with high accuracy (adj. *R* ^*2*^ = 0.55). The combination of cortical thickness and CortBS parameters provided similar or better prediction accuracies of proximal femur stiffness and strength than aBMD.

In this cross‐sectional pilot study, the CortBS method was applied for the first time in humans. Postmenopausal women with and without history of fragility fractures were included. We hypothesized that the frequency‐dependent attenuation and microstructural CortBS parameters can be assessed in vivo and that they are associated with the occurrence of fragility fractures. The in vivo short‐term precision of the CortBS parameters was assessed and the fracture discrimination performance was compared with those of DXA and HR‐pQCT.

## 2 Materials and Methods

### 2.1 Research participants

For this cross‐sectional study, 55 female subjects (aged ≥55 years) who have been referred to the Center for Muscle and Bone Research for a clinically indicated DXA bone density measurement were recruited. Height, weight, age, medical history regarding diseases affecting bone health, chronic diseases, fracture status with differentiation regarding adequate/inadequate trauma, medications negatively affecting bone health, as well as osteoprotective and osteoanabolic medications were assessed. To reflect the distribution of fracture rate with respect to BMD in postmenopausal women, the patient recruitment was stratified into three groups according to the results of the DXA measurement (lowest *T*‐score of lumbar spine and proximal femur) and fracture status, ie, OP: osteoporosis (*T*‐score ≤ −2.5); OPE‐Fx: osteopenia (*T*‐score between −1 and −2.5) and prevalent fragility fracture; OPE‐nFx: osteopenia (*T*‐score between −1 and −2.5) without prevalent fragility fracture. Exclusion criteria were (i) body mass index (BMI) >30; (ii) presence of metal implants or edema at the lower extremity; (iii) no allowance for X‐ray exposure; or (iv) the inability to understand the nature of the study and follow the instructions. In addition to the measurements on patients, repeated ultrasound readings were performed on three healthy volunteers. The study was registered in the German Clinical Trial Register (DRKS00022217) and was approved by the local ethics committee of the Charité–University Hospital Berlin (reference number: EA4/068/19) and the German Federal Office for Radiation Protection (reference number: Z5‐22464/2019‐090‐G). All participants provided their informed written consent before participation.

### 2.2 DXA bone densitometry

DXA (Lunar Prodigy Advance EnCore Software v. 13.4 or Lunar iDXA EnCore Software v. 16.1, GE Medical Systems, Madison, WI, USA) lumbar spine (L_1_ to L_4_) and proximal femur scans were performed as part of the clinical routine examination according to the standard GE Lunar operator manual. The leg (left or right side) with the lowest aBMD at the proximal femur was defined as index leg for subsequent ultrasound and HR‐pQCT readings. aBMD values were assessed at the femoral neck (aBMD_Femur**(**Neck**)**_), the total proximal femur area (aBMD_Femur**(**Total**)**_), and at the spine (aBMD_Spine_). *T*‐scores were calculated, whereas the minimum scores at femur (*T*‐score_Femur_), spine *T*‐score_Spine_, and the lowest value of both anatomical regions (*T*‐score_Total_) were used for further analyses. If the *T*‐score from one region could not be assessed, the other one was defined as *T*‐score_Total_.

### 2.3 Cortical backscatter (CortBS)

**Fig 1:**
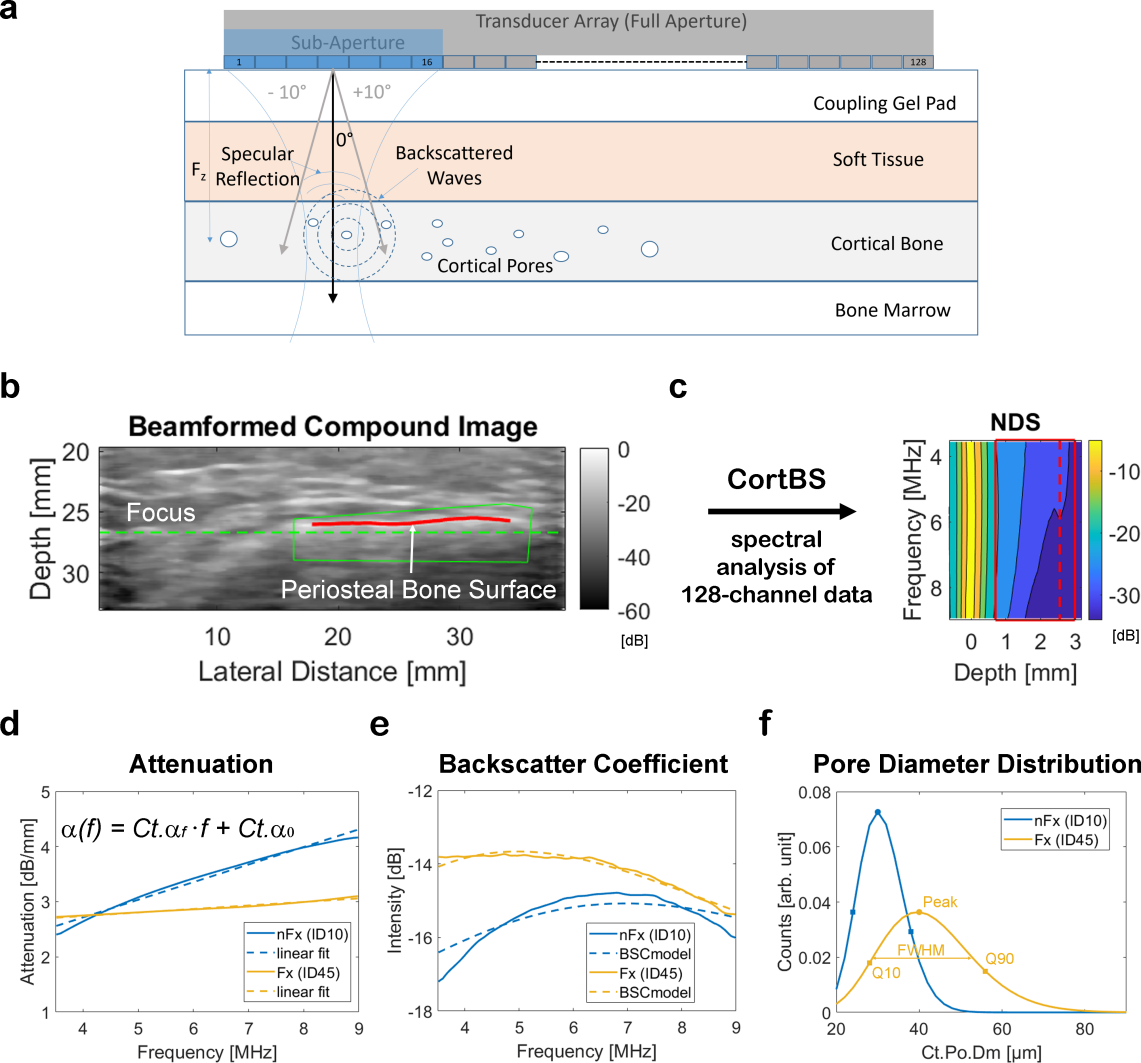
Schematic drawing of the CortBS method (*A*). A focused beam generated by a 16‐element subaperture of the 128‐element transducer array is scanned and steered across the bone. The focus depth F_z_ is positioned approximately 1 mm below the bone surface. Pulse‐echo signals are recorded simultaneously with all 128 channels. The reconstructed compound B‐mode compound image (*B*) shows the anteromedial cross section of the tibia midshaft (green dashed line: focus position; green line: manually selected ROI; red line: detected periosteal interface within ROI). The reconstructed 3D bone surface (red line) is used to calculate a depth‐dependent spectrogram. Spectra arising from specular reflections at the bone surface are used for normalization. From the normalized depth‐dependent backscatter spectrum (NDS) (*C*), the depth and frequency ranges of 1 to 3 mm and 4 to 9 MHz, respectively, are used to derive the attenuation and backscatter coefficients α(f) (*D*) and BSC(f) (*E*). By fitting model‐based backscatter coefficients^(^ ^26^ ^)^ to the measured BSC(f), the cortical pore diameter distribution Ct.Po.Dm.D is estimated (*F*). (*E*, *F*) Shown are representative α(f), BSC(f), and Ct.Po.Dm.D data for one subject with (ID45; *T*‐score_Total_ = −2.0) and one without fragility fractures (ID10; *T*‐score_Total_ = −3.5).

The measurement principle has been described in detail previously.^(^ ^26^ ^)^ Briefly, a medical ultrasound scanner SonixTOUCH equipped with a SonixDAQ single‐channel data acquisition system and a 4DL14‐5/38 3‐D linear array transducer (Ultrasonix, Richmond, Canada) was used. The system was controlled through a custom‐developed user interface. Measurements were performed at the central anteromedial tibia region. The tibia length (L_Tibia_) was assessed as the distance between the medial knee joint cleft and the medial malleolus. Both landmarks were palpated manually. Between these two points, the level of 50% L_Tibia_ was marked with a skin marker pencil. The ultrasound transducer was coupled to the skin at this position using an ultrasound coupling pad (aquaflex, Parker Laboratories, Inc., Fairfield, NJ, USA). Conventional B‐mode images were used to position the probe such that a cross‐sectional image of the periosteal tibia bone interface appeared in the center of the image. The probe was then manually tilted until the bone surface was approximately normal to the sound beam direction and the focus position F_z_ was adjusted to be approximately 1 mm below the periosteal bone surface (Fig. 1*A*).

For the measurement, a compound B‐mode volume scan sequence was used. For the compound B‐mode scan, a slightly focused beam produced by a 16‐element aperture was scanned across the array from element position 1 to 128 with an increment of 1. For each transmit beam, pre‐beamformed pulse‐echo data were acquired from all 128 elements of the probe. The scan was repeated three times with different beam steering angles (−10°, 0°, 10°). The integrated motor allowed one to sweep the transducer array perpendicular to the compound B‐mode imaging plane with tilt angles between ±7° with an increment of 1°. Thereby, a cortical bone surface area of approximately 5 mm × 35 mm was probed at various beam inclination angles. The scan duration was less than 3 seconds. The signal analysis consists of (i) reconstruction of beamformed compound images for all sweep motor positions, ie, spatial compounding of all three beam steering angles (Fig. 1*A*); (ii) manual selection of a region of interest covering the bone region to be analyzed (Fig. 1*B*); (iii) automatic detection and 3D reconstruction of the periosteal bone surface (Fig. 1*B*); (iv) calculation of local beam inclinations, an inclination‐corrected mean surface reflection spectrum and an inclination‐controlled depth‐dependent normalized mean difference spectrum NDS from the pre‐beamformed channel data (Fig. 1*C*); (v) estimation of the frequency‐dependent cortical bone attenuation and backscatter coefficients α(f) and BSC(f), respectively (Fig. 1*D*–*E*); and (vi) the estimation of the cortical pore diameter distribution Ct.Po.Dm.D (Fig. 1*F*). The latter is obtained by minimizing the error between the measured and theoretical BSCs, which are modeled from arbitrary pore‐size distributions. The acquisition and analysis ensure that only signals measured with limited beam inclination (ie, ±10° and ±30° for surface reflection and subsurface backscatter, respectively) were included in the analysis. Except for step 2, all analysis steps were processed fully automatically. A quality parameter, which provides a relative measure of usable data within the selected ROI (ie, data were not discarded by inclination, signal level, and other thresholds), was used as an objective criterion to either accept or reject a measurement. Based on repeated measurement with variable probe tilt, a quality score threshold of 77% was found to produce reproducible results (data not shown). From Ct.α(f), slope and intercept values Ct.α_f_ and Ct.α_o_ were obtained by linear regression (Fig. 1*D*). Characteristic parameters describing the asymmetric pore‐diameter distribution Ct.Po.Dm.D (ie, 10% and 90% quantiles Q10 and Q90, respectively; width, minimum and maximum crossing points of full‐width half‐maximum (FWHM) values, and peak position were derived (Fig. 1*F*).

### 2.4 CortBS short‐term precision

The short‐term precision was evaluated according to Gluer and colleagues^(27^ ^)^ by performing 10 repeated measurements with repositioning between each measurement on three healthy volunteers. Absolute and relative precision values were calculated using Equations (4a) and (5) in Gluer and colleagues,^(^ ^27^ ^)^ respectively.

### 2.5 High‐resolution peripheral computed tomography

**Fig 2:**
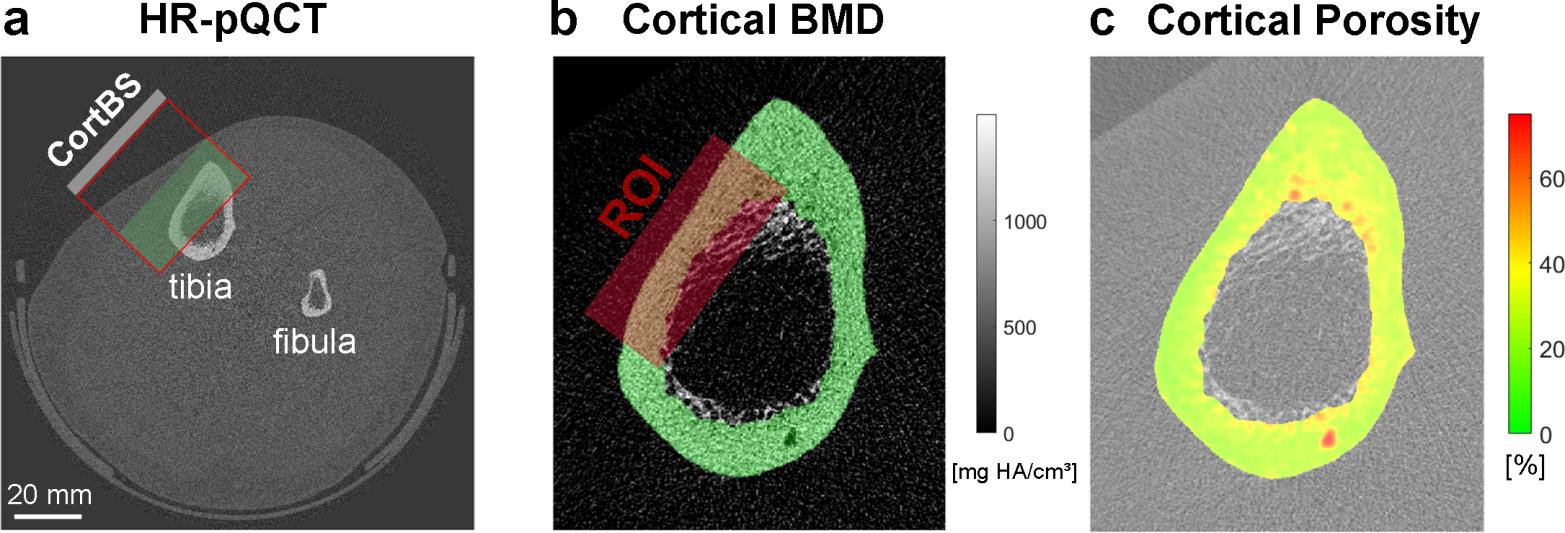
Representative cross‐sectional HR‐pQCT image with the overlaid CortBS measurement region (*A*). The box shaded in green is the image region shown in (*B*). The full tibia cross section and the site‐matched CortBS measurement region were analyzed (*B*). Most of the pores in the cortical bone (marked in green) are unresolved but result in local fluctuations of the voxel values. Pores larger than ~90 μm are resolved. The calculation of a local porosity map (*C*) adapted from Iori and colleagues.^(^ ^20^ ^)^

Immediately after the CortBS measurement, a site‐matched HR‐pQCT scan was performed (XtremeCT II, Scanco Medical AG, Bassersdorf, Switzerland). Subjects were seated in a comfortable, height‐adjustable chair. The lower leg of the subjects was positioned carefully in a carbon‐fiber cast and fixated in the gantry. Subjects were instructed to sit as still as possible and to not talk or move to avoid motion artifacts. The gantry was moved into the scanner until the skin mark and the laser position indicator were aligned. A total scan length of 10.2 mm in the axial direction divided into 168 cross‐sectional images was measured with an isotropic voxel size of 60.7 μm with a scan time of 2 minutes. The total effective dose was less than 5 mSv per scan. A representative reconstructed cross‐sectional image is shown in Fig. 2*A*. Cortical and trabecular properties of the tibia were evaluated using the 3D Density and Structure Analysis software of the scanner as described elsewhere.^(^ ^28^ ^)^ Moreover, cortical properties of (i) the entire tibia cross section (full) and (ii) a manually selected anteromedial region of interest (ROI; Fig. 2) were evaluated using a custom protocol adapted from Iori and colleagues.^(^ ^20^ ^)^ This analysis estimates cortical porosity (Ct.Po_BH_) using the algorithm proposed by Burghardt and colleagues,^(^ ^29^ ^)^ cortical thickness (Ct.Th), pore density (Ct.Po.Dn), and distributions of porosity (Ct.Po.D), pore diameter (Ct.Po.Dm), and bone mineral density (Ct.BMD.D).^(^ ^20^ ^)^ From these distributions, characteristic properties, ie, mean, standard deviation variance, skewness, kurtosis, as well as 10% and 90% quantile (Q10 and Q90) values, were derived.

### 2.6 Statistics

Continuous variables are presented as means and standard deviations (SD). Nonparametric tests were used. Wilcoxon rank sum tests were performed to determine whether parameters were significantly different between the non‐fractured and fractured groups. The correlation between ultrasound and X‐ray parameters was assessed using Spearman's rank sum correlation coefficient ρ. To evaluate the correlation of multiple QUS parameters with HR‐pQCT and DXA parameters, partial least squares (PLS) regression with threefold cross‐validation was used. Spearman's rank sum correlation coefficient ρ and root mean square error (RMSE) between the predicted QUS‐based parameter and those measured by HR‐pQCT or DXA were computed. The fragility fracture discrimination performance of CortBS, HR‐pQCT, and DXA was assessed by means of multivariate PLS discrimination analyses with Leave‐One‐Out Cross‐Validation (PLS‐LOOCV) using the libPLS library.^(^ ^30^ ^)^ For variable selection, a subwindow permutation analysis (SPA) using 10,000 Monte Carlo samplings was repeated until a stable set of significant model variables was found. To avoid overfitting, the final models were created using three PLS components. Different discrimination models were developed to predict vertebral, other, and all fragility fractures from DXA‐based *T*‐scores, HR‐pQCT, or CortBS parameters and for combinations with each subject's anthropometric data (weight, height, BMI), and age. The mean and standard error (SE) of the area under the curve (AUC) of the receiver operation characteristics (ROC), accuracy, sensitivity, and odds ratio (OR) with 95% confidence intervals (CI) were calculated. Differences between the AUC values were evaluated using MedcCalc 20.009a (MedCalc Software Ltd, Ostend, Belgium) by means of two‐sided Hanley & McNeil tests.^(^ ^31^ ^)^ Except for this test and the PLS‐LOOC and SPA analyses, all statistical tests were performed using the Statistics Toolbox of Matlab R2019b (MathWorks, Natick, MA, USA). Statistical results were considered significant for *p* values <0.05.

## 3 Results

### 3.1 Study population

The patient characteristics are summarized in Table 1. Twenty‐nine subjects had at least one fragility fracture. Among the fractured patients, 18 and 21 had vertebral and non‐vertebral fractures, respectively. Age and anthropometric data were not different between fractured (Fx) and non‐fractured (nFx) groups (Table 1). More subjects with fragility fractures received antiresorptive treatment than subjects without fractures. The higher number of subjects treated with an aromatase inhibitor was not significant (*p* = 0.06).

**Table 1:** Age, Basic Anthropometric Data, Disease, and Medication History of the Patient Cohorts With (Fx) and Without (nFx) Fragility Fractures

	All patients (*n* = 55)	Fx (*n* = 29)	nFx (*n* = 26)
Age (years)	69.9 ± 7.2	69.2 ± 7.5	70.2 ± 6.3
Height (cm)	164.2 ± 7.6	165.0 ± 8.1	163.1 ± 7.2
Weight (kg)	62.1 ± 8.3	62.4 ± 9.1	61.0 ± 6.7
Body mass index (kg/m^2^)	23.0 ± 2.8	22.9 ± 2.8	23.0 ± 2.7
Diseases			
Diabetes	3	2	1
Rheumatic diseases	11	7	5
Other chronic inflammatory diseases	4	2	2
Medication			
Antiresorptive	23	17^a^	6
Osteoanabolic	5	4	1
Vitamin D	54	28	26
Selective estrogen receptor modulator (SERM)	3	1	2
Corticosteroid oral	14	7	7
Corticosteroid inhaled	3	2	1
Aromatase inhibitor	6	1	5
Proton pump inhibitor	4	1	3
Other medications^a^	25	15	10

Values are given as mean (SD) or number of subjects.

^a^ *p* < 0.05.

### 3.2 DXA

A valid vertebral spine *T*‐score could not be assessed in 8 subjects because of severe degenerative changes in the lumbar spine. DXA parameters were associated with a subject's height (ρ = 0.65), weight (ρ = 0.60), and almost independent of BMI (ρ = 0.50) and age (ρ = 0.45) (Supplemental Table S1). None of the aBMD values and *T*‐scores were significantly different between Fx and nFx groups (Table 2), but the difference of the lowest total *T*‐scores between fractured and non‐fractured groups almost reached the significance level (*p* = 0.06).

**Table 2:** DXA Range, means, and SDs in Fractured (Fx) and Non‐fractured (nFx) Groups

Parameter	Range	Fx (*n* = 29)	nFx (*n* = 26)
aBMD_Femur(Total)_ (g/cm^2^)	0.668–1.004	0.786 ± 0.072	0.808 ± 0.076
aBMD_Femur(Neck)_ (g/cm^2^)	0.635–0.991	0.793 ± 0.081	0.805 ± 0.072
aBMD_Spine_ (g/cm^2^)^a^	0.651–1.242	0.904 ± 0.130^b^	0.950 ± 0.110^c^
*T*‐score_Femur_	−3.1 to 1.5	−1.93 ± 0.86	−1.83 ± 0.55
*T*‐score_Spine_ ^a^	−4.3 to 0.5	−2.28 ± 1.04^b^	−1.90 ± 0.90^c^
*T*‐score_Total_ ^a^	−4.3 to −1.4	−2.41 ± 0.72^b^	−2.14 ± 0.66^c^

^a^ *n* = 47.

^b^ *n* = 28.

^c^ *n* = 19.

### 3.3 HR‐pQCT

Data from one subject could not be evaluated because of an apparent motion artifact. From the remaining 54 subjects, 81 structure and material properties were extracted. HR‐pQCT parameters obtained from the scanner software were associated with a subject's weight (ρ = 0.68), height (ρ = 0.67), age (ρ = 0.54), and almost independent of BMI (ρ = 0.44). Except for BMI, the associations of cortical parameters derived from the custom analysis with anthropometric data and age were generally lower (Supplemental Table S1). None of the parameters derived from the scanner software were significantly different between fractured and non‐fractured groups (Table 3). In contrast, most parameters describing the local distributions of porosity and pore diameter in the anteromedial region of interest were significantly different between both groups. The most prominent differences were observed for skewness (*p* = 0.004) and kurtosis (*p* = 0.004) of the Ct.Po.Dm.D evaluated in the full cross sections.

**Table 3:** HR‐pQCT Range, Means, and SDs of Selected Parameters in Fractured (Fx) and Non‐fractured (nFx) Groups

	Range	Fx (*n* = 29)	nFx (*n* = 25)
Bone geometry			
Tt.Ar (mm^2^)	313–536	420 ± 56	407 ± 47
Ct.Pm (mm)	72–100	86 ± 7	84 ± 5
Ct.Ar (mm^2^)	170–318	254 ± 31	252 ± 40
Tb.Ar (mm^2^)	97–298	170 ± 47	159 ± 41
Tb.Meta.Ar (mm^2^)	40–121	69 ± 19	64 ± 17
Tb.Inn.Ar (mm^2^)	58–177	101 ± 28	94 ± 25
Bone density			
Tt.vBMD (mg HA/cm^3^)	439–748	596 ± 77	611 ± 65
Tb.vBMD (mg HA/cm^3^)	32–165	77 ± 30	78 ± 30
Tb.Meta.vBMD (mg HA/cm^3^)	106–291	184 ± 51	184 ± 35
Tb.Inn.vBMD (mg HA/cm^3^)	−22 to 109	4.3 ± 20	5.8 ± 30
Ct.vBMD (mg HA/cm^3^)	826–1049	930 ± 53	940 ± 31
Bone structure			
BV/TV	0.07–0.25	0.13 ± 0.04	0.13 ± 0.04
Tb.N (1/mm)	0.5–1.8	1.12 ± 0.29	1.18 ± 0.29
Tb.Th (mm)	0.19–0.36	0.27 ± 0.04	0.27 ± 0.03
Tb.Sp (mm)	0.56–2.05	1.01 ± 0.30	0.94 ± 0.29
Tb.1/N.SD (mm)	0.18–1.31	0.45 ± 0.22	0.39 ± 0.18
Ct.Th (mm)	2.8–6.56	4.96 ± 0.57	4.96 ± 0.83
Ct.Po (%)	0.4–8.2	2.5 ± 1.9	2.0 ± 1.0
Ct.Po.Dm (mm)	0.15–0.33	0.21 ± 0.04	0.22 ± 0.04
Custom (ROI)			
Ct.Th_(ROI)_ (mm)	1.0–4.2	2.7 ± 0.8	2.7 ± 0.6
Ct.Po_BH(ROI)_ (%)	1.1–11.1	5.4 ± 2.3	4.5 ± 2.2
Cortical porosity distribution			
Ct.Po.D_Mean(ROI)_ (%)	14.7–33.8	26.0 ± 4.6	25.3 ± 3.6
Ct.Po.D_SD(ROI)_ (%)	3.7–9.6	**6.0 ± 1.2** ^**a**^	**5.4 ± 1.1**
Ct.Po.D_VAR(ROI)_ (%)	13.5–92.9	**37.5 ± 16.2** ^**a**^	**30.1 ± 13.2**
Ct.Po.D_skewness(ROI)_	0.5–2.9	1.0 ± 0.4	1.4 ± 0.5
Ct.Po.D_skewness(Full)_	0.48–2.93	**0.98 ± 0.37** ^**a**^	**1.35 ± 0.51**
Ct.Po.D_kurtosis(ROI)_	3.1–17.2	5.1 ± 1.5	7.3 ± 3.4
Ct.Po.D_kurtosis(Full)_	3.1–17.2	**5.1 ± 1.5** ^**a**^	**7.3 ± 3.4**
Cortical pore‐diameter distribution			
Ct.Po.Dm.D_Mean(ROI)_ (μm)	96–185	**128 ± 20** ^**a**^	**120 ± 15**
Ct.Po.Dm.D_SD(ROI)_ (μm)	39–165	83 ± 28	74 ± 23
Ct.Po.Dm.D_Q90(ROI)_ (μm)	153–417	**230 ± 54** ^**a**^	**205 ± 35**
Cortical bone BMD distribution			
Ct.BMD.D_kurtosis(Full)_	3.15–5.54	**3.49 ± 0.48** ^**a**^	**3.52 ± 0.23**

Significant differences are marked in bold. Definitions and descriptions of all variables are summarized in Supplemental Table S3.

^a^ *p* < 0.05.

### 3.4 CortBS

An ultrasound compound image of the anteromedial region of the tibia of bone together with the normalized difference spectrum and representative backscatter and attenuation coefficients and pore‐size distributions for subjects with and without fragility fractures are shown in Fig. 1. The short‐term precision of the individual parameter estimations was in the range between 7.9% and 13.9% (Table 4). For 5 patients, the quality factor was below 77% and, therefore, data were not analyzed. CortBS parameters were associated with subject's age (ρ = 0.67), height (ρ = 0.50), and marginally with weight (ρ = 0.45) and BMI (ρ = 0.46) (Supplemental Table S1). Parameter ranges and differences between fractured and non‐fractured groups are summarized in Table 3. Slope Ct.α_f_ and intercept Ct.α_0_ values of the attenuation coefficient were significantly different between fractured and non‐fractured groups. The change of the width of the pore size distribution (Ct.Po.Dm.D_FWHM_) almost reached the significance level (*p* = 0.06).

**Table 4:** CortBS Short‐Term Precision (Absolute and Relative), Range, Mean, and SD Values in Fractured (Fx) and Non‐fractured (nFx) Groups

Parameter	Precision	Range	Fx (*n* = 25)	nFx (*n* = 25)
Ct.α_o_ (dB/mm)	0.22 (13.91)	1.06–3.10	**2.34 ± 0.40** ^a^	**1.96 ± 0.48**
Ct.α_f_ (dB/MHz/mm)	0.02 (15.29)	0.01–0.32	**0.11 ± 0.06** ^a^	**0.16 ± 0.06**
Ct.Po.Dm.D_Peak_ (μm)	2.51 (8.47)	24–52	38.6 ± 5.6	36.6 ± 7.1
Ct.Po.Dm.D_Q10_ (μm)	1.93 (7.89)	20–42	29.9 ± 4.7	28.8 ± 5.8
Ct.Po.Dm.D_Q90_ (μm)	3.28 (8.64	30–64	48.7 ± 7.1	45.3 ± 8.8
Ct.Po.Dm.D_FWHM_ (μm)	1.43 (11.99)	9.4–25.6	16.5 ± 3.5	14.9 ± 2.9
Ct.Po.Dm.D_FWHM,min_ (μm)	2.09 (8.53)	20.0–42.8	31.1 ± 5.0	29.5 ± 5.8
Ct.Po.Dm.D_FWHM,max_ (μm)	3.15 (8.62)	29.4–62.8	47.6 ± 6.8	44.4 ± 8.6

Significant differences are marked in bold.

^a^ *p* < 0.05.

### 3.5 Fragility fracture discrimination

**Fig 3:**
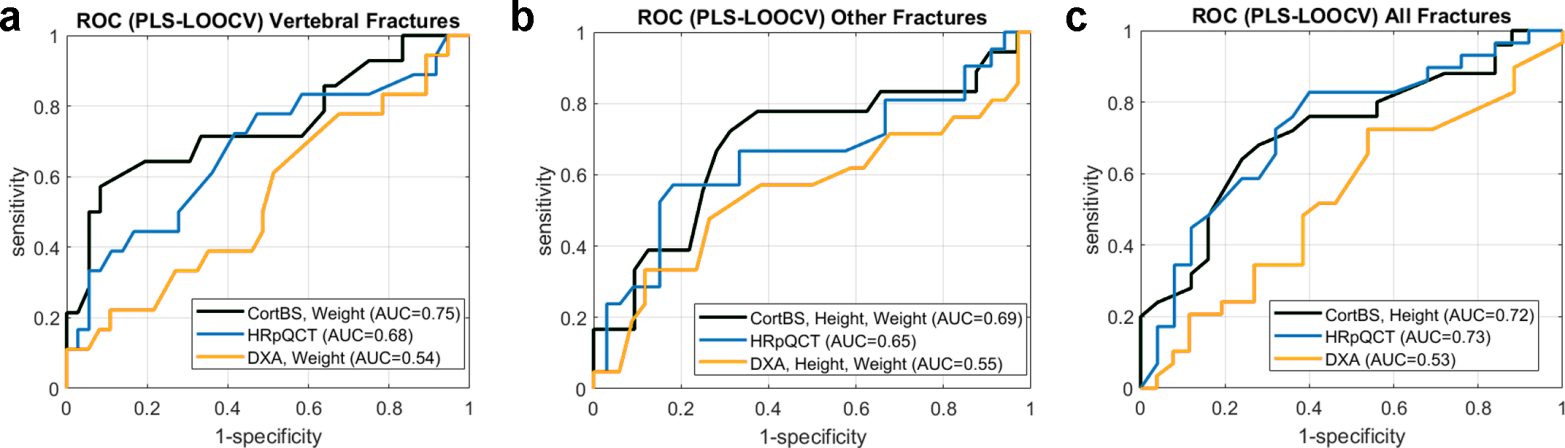
Fragility fracture discrimination performance of DXA, HR‐pQCT, and CortBS for vertebral fractures (*A*), other fractures (*B*), and all fractures (*C*). If anthropometric information improved the discrimination model, these ROC curves are shown.

The results of discrimination performance analyses are summarized in Fig. 3 and Table 5. The DXA‐based *T*‐score values alone did not provide any significant discrimination model. Incorporating a subject's weight and height yielded significant models with, however, poor discrimination performance for vertebral and other fractures (0.54 ≤ AUC ≤ 0.55). Among all HR‐pQCT parameters, those describing the shape distributions of porosity and pore diameter were the most predictive ones. Distinct parameter combinations provided good discrimination models for vertebral, non‐vertebral, and all fragility fractures (0.66 ≤ AUC ≤ 0.73). Age and anthropometric information could not further improve the discrimination models. CortBS parameters provided good discrimination models for all types of fragility fractures (0.65 ≤ AUC ≤ 0.72). Whereas for non‐vertebral fractures only attenuation parameters (Ct.α_0_ and Ct.α_f_) were selected, vertebral and all fractures were discriminated by a combination of attenuation and pore‐size distribution parameters. Incorporation of weight and height information led to non‐significant increases of the AUC values.

**Table 5:** Fragility Fracture Discrimination Performance: PLS‐LOOC Discrimination Models Were Developed for the Individual Measurement Modalities Alone and in Combination With Anthropometric (AP) Data and Age

	Sensitivity	Specificity	AUC (SE)	Accuracy	OR (95% CI)	Variables
Vertebral fractures (Fx/nFx)						
DXA + AP (11/36)	0.11	0.94	0.54 (0.006)	0.67	2.2 (0.1–4.2)	*T*‐score_Femur_ Weight
HR‐pQCT (18/36)	0.27	0.92	0.68^a^ (0.03)	0.70	4.2 (2.7–5.8)	Ct.Po.D_SD(ROI)_ Ct.Po.D_VAR(ROI)_ Ct.Po.Dm.D_Mean(ROI)_ Ct.Po.Dm.D_SD(ROI)_ Ct.Po.Dm.D_Q90(ROI)_
CortBS (14/36)	0.43	0.92	0.72^a^ (0.009)	0.78	8.2 (6.7–9.8)	Ct.α_0_, Ct.α_f_ Ct.Po.Dm.D_FWHM_ Ct.Po.Dm.D_FWHM‐Max_ Ct.Po.Dm.D_Q90_
CortBS + AP (14/36)	0.50	0.94	0.75^a^ ^,^ ^b^ (0.01)	0.82	17.0 (15.2–18.8)	Ct.α_0_, Ct.α_f_ Ct.Po.Dm.D_FWHM_ Ct.Po.Dm.D_Q90_ Weight
Other fractures (Fx/nFx)						
DXA + AP (15/34)	0.33	0.85	0.55 (0.02)	0.65	2.9 (1.6–4.2)	*T*‐score_Femur_ Height, weight
HR‐pQCT (15/33)	0.48	0.85	0.66^a^ (0.03)	0.70	5.1 (3.8–6.4)	Ct.Po_BH(ROI)_ Ct.Po.D_skewness(ROI)_ Ct.Po.Dn_(ROI)_ Ct.Po_BH(Full)_ Ct.Po.D_skewness(Full)_ Ct.Po.D_kurtosis(Full)_ T.Tb.Th_(Full)_
CortBS (12/32)	0.39	0.81	0.65^a^ (0.007)	0.66	2.76 (1.5–4.1)	Ct.α_0_, Ct.α_f_
CortBS + AP (12/32)	0.39	0.88	0.69^a^ (0.02)	0.70	4.45 (3.0–5.9)	Ct.α_0_, Ct.α_f_ Height, weight
All fractures (Fx/nFx)						
HR‐pQCT (29/26)	0.83	0.64	0.73 (0.005)	0.74	8.5 (7.3–9.8)	Ct.Po.D_Q90(ROI)_ Ct.Po.D_skewness(Full)_
CortBS (29/25)	0.68	0.64	0.69 (0.02)	0.66	3.8 (2.6–4.9)	Ct.α_0_, Ct.α_f_ Ct.PoDm.D_Q10_ Ct.PoDm.D_Q90_ Ct.PoDm.D_Peak_ Ct.PoDm.D_FWHM‐Min_ Ct.PoDm.D_FWHM‐Max_
CortBS + AP (29/25)	0.72	0.64	0.72 (0.006)	0.68	4.6 (3.4–5.8)	Ct.α_0_, Ct.α_f_ Height

Only significant models are listed. The numbers of fractured/non‐fractured cases for each model are written in the first column in parentheses. Significant variables selected by SPA are listed in the last column. Significant differences of the AUC values between the models for each fracture group are indicated by superscript letters.

^a^ AUC(CortBS/HRpQCT) > AUC(DXA + AP).

^b^ AUC(CortBS+AP) > AUC(HR‐pQCT).

### 3.6 Associations between HR‐pQCT and CortBS parameters

Attenuation was not associated with bone geometry except for one weak correlation between Ct.α_0_ and Ct.Th (Supplemental Table S2). Multiple univariate associations were found for attenuation and Ct.Po.Dm.D parameters with bone density, structure, and porosity, and pore‐diameter distributions. Most HR‐pQCT parameters could be predicted with weak to moderate accuracy (0.28 ≤ ρ ≤ 0.71) by combinations of CortBS parameters.

### 3.7 Ultrasound‐based BMD prediction

**Fig 4:**
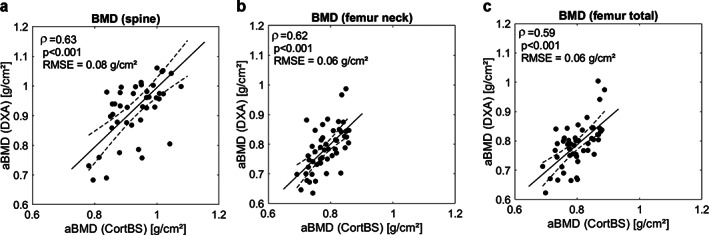
Estimation of aBMD at spine (*A*), femur neck (*B*), and total proximal femur (*C*) from ultrasound backscatter and anthropometric parameters using PLS regression.

Fig. 4 shows the prediction of aBMD from CortBS parameters using multivariate PLS models. Although significant, the correlations were moderate (0.59 ≤ ρ ≤ 0.63).

## 4 Discussion

The diagnosis of osteoporosis based on the assessment of structural deterioration of the porous micromorphology has been prevented by the limitations of currently available diagnostic technologies.^(^ ^7^ ^)^ The established diagnosis of OP based on aBMD captures the relative bone loss of an individual compared with a mean value of a young reference population but not the individual bone loss caused by impaired bone remodeling. Thereby, people with non‐pathologically decreased *T*‐scores but at risk for fragility fractures are currently undiagnosed until the fracture occurs.^(^ ^8^ ^)^ Alternative diagnostic modalities provide X‐ray radiation‐free BMD and *T*‐score surrogates^(^ ^32^, ^33^, ^34^, ^35^ ^)^ but do not overcome the principal lack of sensitivity of BMD to the causal microstructural and tissue deteriorations caused by OP. To date, HR‐pQCT is the most sophisticated in vivo imaging modality for the microstructural analysis of bone. It can resolve pathologically enlarged cortical pores, but the spatial resolution is not sufficient to resolve the normal cortical pore micromorphology.^(^ ^20^ ^)^ In this work, we have applied for the first time in humans a novel ultrasound technology that provides noninvasively and without ionizing radiation quantitative information about the pore‐size distribution together with frequency‐dependent attenuation in cortical bone at the tibia midshaft. The fracture discrimination performance of the novel CortBS technology was compared against conventional DXA‐based diagnosis and state‐of‐the‐art X‐ray computed tomography (HR‐pQCT).

### 4.1 The anteromedial tibia is a favorable site for the assessment of systemic structural bone tissue deteriorations leading to fragility fractures

The standard locations for DXA measurements (ie, L_1_ to L_4_ lumbar spine and hip and forearm) were initially selected because morbidity from fractures at these locations is high.^(^ ^7^ ^)^ However, metal implants, previous fractures, scoliosis, osteosclerosis, and aortic calcifications render aBMD estimations at these sites inaccurate or even impossible.^(^ ^36^ ^)^ The most standardized HR‐pQCT measurement site is the distal tibia.^(^ ^28^ ^)^ In an ex vivo study, hip failure load has been reported to be associated with low vBMD and microstructural alterations measured at this site.^(^ ^37^ ^)^ However, toward the epiphyses, cortical bone becomes thinner and is increasingly replaced by a trabecular core. Therefore, parameters assessed in this region are susceptible to both positioning errors and inter‐subject anatomical differences. In this study, we have used the tibia midshaft region for the following reasons. First, the midshaft contains predominantly cortical bone. Second, the cortical thickness in the anteromedial measurement midshaft region is relatively invariant with respect to the long‐axis position and approximately two to three times larger^(^ ^17^ ^)^ than reference values reported for the proximal tibia.^(^ ^38^ ^)^ Thereby, the tibia midshaft provides a much larger and anatomically more invariant tissue volume for cortical bone microstructural characterization than the distal shaft. Recent ex vivo studies provided evidence that structural deterioration at this measurement site is associated with reduced proximal femur strength^(^ ^17^ ^)^ and that the parameters assessed by CortBS combined with Ct.Th provide superior predictions of proximal femur stiffness and strength compared with aBMD.^(^ ^26^ ^)^ Ultrasound can be transmitted most easily to and along bone at the facies medialis of the tibia midshaft, where the periosteum is covered by a thin layer of soft tissue only. Already in 1995, Foldes and colleagues^(^ ^39^ ^)^ suggested speed of sound measured by axial transmission at the tibia midshaft as an independent predictor of fracture risk in women with non‐osteoporotic bone mineral density.^(^ ^39^ ^)^ Since then, various novel bone QUS techniques have targeted this site for the measurement of Ct.Th, Ct.Po,^(^ ^40^, ^41^ ^)^ and speed of sound.^(^ ^42^ ^)^ This study confirmed that a compromised pore architecture of the cortical tibia midshaft is associated with bone fragility. In line with the well‐known microstructural deteriorations induced by OP, both HR‐pQCT and CortBS revealed predominantly features describing the asymmetry of the cortical pore‐size distribution rather parameters describing the mean pore‐tissue volume fraction as factors associated with fragility fractures. Moreover, frequency‐dependent ultrasound attenuation, which is determined by both structural and viscoelastic tissue properties,^(26^ ^)^ was found to be significantly altered in subjects with fragility fractures. Recent numerical ultrasound transmission studies on three‐dimensional bone mimicking structures suggested that pore radius and density can be inferred from the frequency dependence of ultrasonic attenuation.^(^ ^43^ ^)^ In that study, monodisperse pore radii ranging from 50 to 100 μm and densities ranging from 20 to 50 pores per mm^3^ were investigated. The same group also proposed a model that aims at decoupling the effects of viscoelastic absorption and scattering.^(^ ^44^ ^)^ Although forward and backscatter characteristics are not identical, the same concept could be integrated into the cortical backscatter model in the future to assess the relative contributions of structural and viscoelastic tissue alterations to the fragility fracture discrimination independently.

### 4.2 Discrimination performance

The results of this pilot study suggest a superior discrimination performance of the ultrasonic cortical backscatter measurement (0.69 ≤ AUC ≤ 0.75) compared with DXA (0.54 ≤ AUC ≤ 0.55) and a similar or even better performance compared with HR‐pQCT (0.66 ≤ AUC ≤ 0.73). The two attenuation parameters Ct.α_0_ and Ct.α_f_ were the strongest predictors for all types of fragility fractures. Together with the subject's height and weight, cortical bone attenuation provided the best discrimination performance for non‐vertebral fractures (AUC = 0.69). The subject's height is a known risk factor for non‐vertebral fractures,^(^ ^45^ ^)^ which has been partly linked to thinner and more porous cortices in taller women, as measured at the distal tibia by first‐generation HR‐pQCT.^(^ ^46^ ^)^ Ct.Th at the tibia midshaft was not a predictive variable in our study, but both HR‐pQCT and CortBS measurements confirmed that porosity and pore‐size distributions as well as the mean porosity were associated with fragility fractures.

For vertebral fractures, attenuation together with width and 90% quantile values of the pore‐diameter distribution were significant ultrasound predictor variables, while the subject's weight remained the only anthropometric factor (AUC = 0.75). This finding is in agreement with a previous report suggesting risk factors, eg, physical weakness, poor health, and weight loss, as risk factors for vertebral but not for non‐vertebral fractures.^(^ ^47^ ^)^

Our AUC values were lower for DXA and comparable for QUS parameters than those reported in another study, in which cortical thickness and porosity were estimated from axial transmission ultrasound.^(^ ^41^ ^)^ Although in that study on 201 postmenopausal women Ct.Th in was found to be discriminant for hip fractures only (AUC = 0.72), Ct.Po was discriminant for all fractures (AUC = 0.71) and for vertebral (AUC = 0.84) and wrist fractures (AUC = 0.71).

Several bone QUS technologies have been used in the past to measure cortical or cancellous bone sites, and at least some of them have demonstrated the potential to predict fracture risk with an equivalent efficiency compared with X‐ray densitometry techniques.^(^ ^23^, ^48^ ^)^ Although ultrasound wave propagation is governed by the structural and material properties of the propagation medium, none of the currently available clinical devices provide any direct measurement of stiffness, strength, or tissue quality. Instead, they provide bone density, stiffness, or quality surrogate markers derived from empirical correlations of acoustic properties (eg, speed of sound [SOS] and broadband ultrasound attenuation [BUA]),^(^ ^23^ ^)^ travel time delays,^(^ ^33^, ^49^ ^)^ or the shape of the backscatter spectrum^(^ ^35^ ^)^ with aBMD. For example, Adami and colleagues^(^ ^50^ ^)^ used *T*‐scores derived from radiofrequency echographic multi spectrometry (REMS) in comparison with DXA‐based *T*‐scores for the discrimination of women with and without fractures as the identification of patients at risk for incident osteoporotic fractures. This prospective study on 1516 white women (aged 30 to 90 years) reported similar prediction performance for DXA‐ and QUS‐based *T*‐scores. A model‐based measurement of Ct.Th and Ct.Po in radius and tibia bones has been achieved for the first time with the bidirectional axial transmission technology by means of multimode waveguide dispersion analysis.^(^ ^40^, ^51^ ^)^ The method considers variations of porosity as a major source of variations of cortical bone elasticity, sound velocity, and fracture toughness in postmenopausal women.^(^ ^52^, ^53^, ^54^ ^)^ Results of a first validation study in postmenopausal women confirmed a comparable fracture discrimination performance of the BDAT variables as aBMD for both vertebral and peripheral fractures.^(^ ^41^ ^)^ However, axial transmission measurements do not provide direct image guidance and are restricted to patients with low BMI.

CortBS reflects viscoelastic and microstructural deteriorations of cortical bone, which are causally linked to the natural aging process and the development of osteoporosis.^(^ ^11^ ^)^ The crucial role of the porous microarchitecture, particularly the prevalence of large pores as a biomarker for reduced bone strength,^(17^ ^)^ was also confirmed in the HR‐pQCT analysis, which revealed the asymmetry of the porosity distribution but not the total porosity as a fracture discriminating tissue property. In contrast to that ex vivo study, which included bone from both male and female donors, Ct.Th was not found to be a fracture discriminating biomarker in our study.

### 4.3 Limitations

This pilot study has several limitations. First, the cohort size was small and restricted to postmenopausal women with *T*‐scores below −1. Second, the included subjects had diverse fracture and medication histories as well as various comorbidities. However, the selected cohort resembles the population that is (i) most vulnerable for fragility fractures and (ii) mostly undertreated based on the BMD diagnosis. Despite these limitations, a good discrimination performance was achieved, which needs to be confirmed in larger studies covering a larger age range, both sexes, larger BMI ranges, and *T*‐scores above −1. Third, the cross‐sectional study design did not allow us to assess fracture risk. Future prospective studies should therefore evaluate the potential of CortBS parameters to identify people at risk and to assess the individual fracture risk. Second, no real‐time assessment of the CortBS measurement quality was possible in this study, which led to the exclusion of data from 5 subjects during the post hoc data analysis. For clinical applications, the data‐quality assessment needs to be incorporated into the measurement, providing real‐time feedback to the operator and the possibility to repeat the measurement, until an appropriate data quality is achieved.

CortBS is the first quantitative bone imaging modality that can quantify microstructural tissue deteriorations in cortical bone, which occur during normal aging and the development of osteoporosis. CortBS discriminates fragility fractures in postmenopausal women better than aBMD. It could be used as a portable, low‐cost, non‐ionizing, and widely applicable screening tool to identify people at risk, particularly in the population with low bone mass.

## Disclosures

JM is an employee of poroUS GmbH, a startup developing the CortBS technology. KR is the inventor on the patent applications (EP3641657A1, US 2020/0129140, CN110769754A, and JP 2019‐570514) describing the CortBS technology.

5

### Peer review

The peer review history for this article is available at https://publons.com/publon/10.1002/jbm4.10536.

## Supporting information


**Supplemental Table S1**. Associations between DXA, selected HR‐pQCT, and CortBS parameters with anthropometric data and age. For HR‐pQCT, either the parameters derived from the vendors “3D Density and Structure Analysis” (3D DSA) or the custom parameters selected from the fracture discrimination analysis (Table 5) were used. The values show the Spearman's rank sum correlation coefficient ρ.
**Supplemental Table S2**. Associations between selected site‐matched HR‐pQCT (ROI) and CortBS parameters. The values show the Spearman's rank sum correlation coefficient ρ. The last rows show ρ and RMSE obtained from the multivariate PLS.
**Supplemental Table S3**. Definition, units, and description of the HR‐pQCT parameters derived from the reported in Table 3.
