# Supplementary material for: Pore‐Size Distribution and Frequency‐Dependent Attenuation in Human Cortical Tibia Bone Discriminate Fragility Fractures in Postmenopausal Women With Low Bone Mineral Density
Source: JBMR Plus. 2021 Sep 2;5(11):e10536. doi: 10.1002/jbm4.10536 (PMC8567489; doi:10.1002/jbm4.10536)
Supplement: Supplementary file 1 — Supplemental Table S1. Associations between DXA, selected HR‐pQCT, and CortBS parameters with anthropometric data and age. For HR‐pQCT, either the parameters derived from the vendors “3D Density and Structure Analysis” (3D DSA) or the custom parameters selected from the fracture discrimination analysis (Table 5) were used. The values show the Spearman's rank sum correlation coefficient ρ. Supplemental Table S2. Associations between selected site‐matched HR‐pQCT (ROI) and CortBS parameters. The values show the Spearman's rank sum correlation coefficient ρ. The last rows show ρ and RMSE obtained from the multivariate PLS. Supplemental Table S3. Definition, units, and description of the HR‐pQCT parameters derived from the reported in Table 3. [file JBM4-5-e10536-s001.docx]

| **Table A.1 \| Associations between DXA, selected HR-pQCT, and CortBS parameters with anthropometric data and age. For HR-pQCT, either the parameters derived from the vendors “3D Density and Structure Analysis” (3D DSA) or the custom parameters selected from the fracture discrimination analysis (Table 5) were used. The values show the Spearman’s rank sum correlation coefficient ρ.** | | | | |
| --- | --- | --- | --- | --- |
|  | **Weight** | **Height** | **BMI** | **Age** |
| **DXA** | 0.60 | 0.65 | 0.50 | 0.45 |
| **HR-pQCT(3D DSA)** | 0.68 | 0.67 | 0.44 | 0.54 |
| **HR-pQCT(custom)** | 0.61 | 0.58 | 0.44 | 0.52 |
| **CortBS** | 0.45 | 0.50 | 0.46 | 0.67 |

| Table A.2 \| Associations between selected site-matched HR-pQCT (ROI) and CortBS parameters. The values show the Spearman’s rank sum correlation coefficient ρ. The last rows show ρ and RMSE obtained from the multivariate PLS. | | | | | | | | | | | | |
| --- | --- | --- | --- | --- | --- | --- | --- | --- | --- | --- | --- | --- |
|  | | **Attenuation** | | **Ct.Po.Dm.D** | | | | | | **PLS** | | |
|  | | α_0_ | α_f_ | Peak | Q_10_ | Q_90_ | FWHM | FWHM_Min_ | FWHM_Max_ | ρ | RMSE | |
| Bone Geometry | | | | | | | | | | | | |
| *Tt.Ar* [mm²] | | - | - | - | 0.28 | - | - | 0.30 | - | 0.51 | 44 | |
| *Ct.Pm* [mm] | | - | - | 0.33 | 0.33 | 0.31 | - | - | - | 0.48 | 5.1 | |
| *Ct.Ar* [mm²] | | - | - | - | - | - | - | - | - | 0.57 | 30.0 | |
| *Tb.Ar* [mm²] | | - | - | 0.31 | 0.34 | 0.30 | - | 0.33 | 0.30 | 0.40 | 37.8 | |
| *Tb.Meta.Ar* [mm²] | | - | - | 0.32 | 0.34 | 0.30 | - | 0.34 | 0.31 | 0.43 | 15.2 | |
| *Tb.Inn.Ar* [mm²] | | - | - | 0.31 | 0.33 | 0.29 |  | 0.33 | 0.30 | 0.43 | 22.1 | |
| Bone Density | | | | | | | | | | | | |
| *Tt.vBMD* [mg HA/cm³] | | - | - | -0.32 | -0.35 | -0.32 | - | -0.34 | -0.32 | 0.55 | | 59 |
| *Tb.vBMD* [mg HA/cm³] | | 0.41 | -0.44 | 0.41 | 0.42 | 0.45 | 0.45 | 0.38 | 0.45 | 0.64 | | 24 |
| Tb.Meta.vBMD [mg HA/cm³] | | 0.45 | -0.51 | 0.47 | 0.49 | 0.50 | 0.48 | 0.44 | 0.50 | 0.71 | | 29 |
| *Tb.Inn.vBMD* [mg HA/cm³] | | 0.31 | - | - | - | 0.28 | - | - | 0.28 | 0.36 | | 21 |
| *Ct.vBMD* [mg HA/cm³] | | -0.53 | 0.45 | -0.46 | -0.49 | -0.49 | -0.45 | -0.49 | -0.49 | 0.69 | | 32 |
| Bone Structure | | | | | | | | | | | | |
| *BV/TV* | | 0.43 | -0.47 | 0.43 | 0.44 | 0.48 | 0.48 | 0.40 | 0.48 | 0.65 | | 0.03 |
| *Tb.N* [1/mm] | | - | - | - | - | - | - | - | - | 0.28 | | 0.29 |
| *Tb.Th* [mm] | | 0.29 | -0.33 | 0.42 | 0.45 | 0.43 | 0.40 | 0.40 | 0.44 | 0.61 | | 0.03 |
| *Tb.Sp* [mm] | | - | - | - | - | - | - | - | - | 0.30 | | 0.25 |
| *Tb.1/N.SD* [mm] | | - | - | - | - | - | - | - | - | - | | - |
| *Ct.Th* [mm] | | - | - | - | - | - | - | - | - | 0.37 | | 0.61 |
| *Ct.Po* [%] | | 0.45 | -0.43 | 0.46 | 0.55 | 0.44 | 0.37 | 0.51 | 0.46 | 0.69 | | 1.2 |
| *Ct.Po.Dm* [mm] | | - | - | - | - | - | - | - | - | 0.57 | | 0.03 |
| Custom (ROI) | | | | | | | | | | | | |
| *Ct.Th*_(ROI)_ [mm] | | -0.29 | - | - | -0.33 | - | - | -0.28 | - | 0.59 | | 0.54 |
| *Ct.Po*_BH(ROI)_ [%] | | 0.46 | -0.43 | 0.37 | 0.38 | 0.36 | 0.31 | 0.37 | 0.36 | 0.68 | | 1.7 |
|  | Cortical Porosity Distribution | | | | | | | | | | | |
| *Ct.Po.D*_Mean(ROI)_ [%] | | 0.40 | -0.32 | 0.31 | 0.33 | 0.29 | - | 0.34 | 0.29 | 0.57 | | 3.2 |
| *Ct.Po.D*_SD(ROI)_ [%] | | 0.36 | -0.55 | 0.39 | 0.43 | 0.40 | 0.39 | 0.41 | 0.41 | 0.54 | | 1.1 |
| *Ct.Po.D*_VAR(ROI)_ [%] | | 0.36 | -0.55 | 0.39 | 0.43 | 0.40 | 0.39 | 0.41 | 0.41 | 0.56 | | 13.8 |
| *Ct.Po.D*_skewness(ROI)_ | | - | - | - | - | - | - | - | - | 0.58 | | 0.39 |
| *Ct.Po.D*_skewness(Full)_ | | - | - | - | - | - | - | - | - | 0.58 | | 0.44 |
| *Ct.Po.D*_kurtosis(ROI)_ | | - | - | - | - | - | - | - | - | 0.54 | | 2.1 |
| *Ct.Po.D*_kurtosis(Full)_ | | - | - | - | - | - | - | - | - | 0.47 | | 2.7 |
|  | Cortical Pore Diameter Distribution | | | | | | | | | | | |
| *Ct.Po.Dm.D*_Mean(ROI)_ [µm] | | 0.49 | -0.61 | 0.49 | 0.51 | 0.52 | 0.53 | 0.49 | 0.53 | 0.58 | | 15.3 |
| *Ct.Po.Dm.D*_SD(ROI)_ [µm] | | 0.43 | -0.60 | 0.49 | 0.51 | 0.51 | 0.52 | 0.47 | 0.52 | 0.55 | | 24 |
| *Ct.Po.Dm.D*_Q90(ROI)_ [µm] | | 0.50 | -0.59 | 0.46 | 0.47 | 0.48 | 0.50 | 0.45 | 0.49 | 0.47 | | 42 |
|  | Cortical Bone BMD Distribution | | | | | | | | | | | |
| *Ct.BMD.D*_kurtosis(Full)_ | | - | - | - | - | - | - | - | - | 0.29 | | 0.31 |

| Table A.3 \| Definition, units, and description of the HR-pQCT parameters derived from the reported in Table 3. | | |
| --- | --- | --- |
| Abbreviation [unit] | Definition | Description |
| *Tt.Ar* [mm²] | Total area | Measure of total cross-sectional area within the periosteal surface |
| *Ct.Pm* [mm] | Cortical Perimeter | Length of periosteal perimeter |
| *Ct.Ar* [mm²] | Cortical area | Measure of the total cross-sectional area within the cortical bone compartment |
| *Tb.Ar* [mm²] | Trabecular area | Cross-sectional area of the trabecular compartment |
| *Tb.Meta.Ar* [mm²] | Trabecular area in the meta region | Outer 40% of the cross-sectional area of the trabecular compartment |
| *Tb.Inn.Ar* [mm²] | Trabecular area in the inner region | Inner 60% of the cross-sectional area of the trabecular compartment |
| Bone Density |  |  |
| *Tt.vBMD* [mg HA/cm³] | Total volumetric bone mineral density | Average mineral density within the periosteal surface |
| *Tb.vBMD* [mg HA/cm³] | Trabecular volumetric bone mineral density | Average mineral density within the trabecular compartment |
| *Tb.Meta.vBMD* [mg HA/cm³] | Trabecular volumetric bone mineral density in the meta region | Average mineral density within the outer 40 % of the trabecular compartment |
| *Tb.Inn.vBMD* [mg HA/cm³] | Trabecular volumetric bone mineral density in the inner region | Average mineral density within the inner 40 % of the trabecular compartment |
| *Ct.vBMD* [mg HA/cm³] | Cortical bone mineral density | Average mineral density within the cortical compartment |
| Bone Structure |  |  |
| *BV/TV* | Trabecular bone volume fraction | Ratio of trabecular bone mineral density and 1200 mg HA/cm³ |
| *Tb.N* [1/mm] | Trabecular Number | Average number of trabeculae per unit length |
| *Tb.Th* [mm] | Trabecular thickness | Average thickness of trabeculae |
| *Tb.Sp* [mm] | Trabecular separation | Average distance between trabeculae |
| *Tb.1/N.SD* [mm] | Inhomogeneity of trabecular network | Standard deviation of 1/Tb.N |
| *Ct.Th* [mm] | Cortical thickness | Average thickness of the cortical compartment |
| *Ct.Po* [%] | Cortical porosity | Total volume of cortical pores |
| *Ct.Po.Dm* [mm] | Cortical pore diameter | Average 3D diameter of the pore volumes |
| Custom |  |  |
| *Ct.Th* [mm] | Cortical thickness | Most frequent minimum distance  between peri- and endosteal surfaces ^(17)^ |
| *Ct.Po*_BH_ [%] | Cortical porosity | Average porosity calculated using the Burghardt algorithm ^(29)^ |
| *Ct.Po.D* [%] | Cortical porosity distribution | Histogram of the local porosity estimates using the Iori algorithm ^(20)^ |
| *Ct.Po.Dm.D* [µm] | Cortical pore diameter distribution | Histogram of pore diameters |
| *Ct.vBMD.D* | Cortical vBMD distribution | Histogram of volumetric BMD |
